# L-citrulline supplementation reverses the impaired airway relaxation in neonatal rats exposed to hyperoxia

**DOI:** 10.1186/1465-9921-13-68

**Published:** 2012-08-07

**Authors:** Ramadan B Sopi, Syed IA Zaidi, Mitko Mladenov, Hazbije Sahiti, Zahide Istrefi, Icko Gjorgoski, Azem Lajçi, Muharrem Jakupaj

**Affiliations:** 1 Department of Pharmacy-Biology, Faculty of Medicine, University of Prishtina, St. Martyrs’ Boulevard n.n., Prishtina, 10000, Kosovo; 2 Institute of Biology, Faculty of Natural Sciences and Mathematics, “Sts, Cyril and Methodius” University, Skopje, 1000, Macedonia; 3 Department of Chemical Engineering, Case Western Reserve University, Cleveland, OH, 44106, USA; 4 Department of Chemical Engineering, Case Western Reserve University, Cleveland, OH, 44106, USA

**Keywords:** Airway relaxation, Airway smooth muscle, Argininosuccinate lyase, Argininosuccinate synthase, L-arginine, Nitric oxide, Nitric oxide synthase

## Abstract

**Background:**

Hyperoxia is shown to impair airway relaxation via limiting L-arginine bioavailability to nitric oxide synthase (NOS) and reducing NO production as a consequence. L-arginine can also be synthesized by L-citrulline recycling. The role of L-citrulline supplementation was investigated in the reversing of hyperoxia-induced impaired relaxation of rat tracheal smooth muscle (TSM).

**Methods:**

Electrical field stimulation (EFS, 2–20 V)-induced relaxation was measured under *in vitro* conditions in preconstricted tracheal preparations obtained from 12 day old rat pups exposed to room air or hyperoxia (>95% oxygen) for 7 days supplemented with L-citrulline or saline (*in vitro* or *in vivo*). The role of the L-citrulline/L-arginine cycle under basal conditions was studied by incubation of preparations in the presence of argininosuccinate synthase (ASS) inhibitor [α-methyl-D, L-aspartate, 1 mM] or argininosuccinate lyase inhibitor (ASL) succinate (1 mM) and/or NOS inhibitor [N^ω^-nitro-L-arginine methyl ester; 100 μM] with respect to the presence or absence of L-citrulline (2 mM).

**Results:**

Hyperoxia impaired the EFS-induced relaxation of TSM as compared to room air control (p < 0.001*;* 0.5 ± 0.1% at 2 V to 50.6 ± 5.7% at 20 V in hyperoxic group: 0.7 ± 0.2 at 2 V to 80.0 ± 5.6% at 20 V in room air group). Inhibition of ASS or ASL, and L-citrulline supplementation did not affect relaxation responses under basal conditions. However, inhibition of NOS significantly reduced relaxation responses (p < 0.001), which were restored to control level by L-citrulline. L-citrulline supplementation *in vivo* and *in vitro* also reversed the hyperoxia-impaired relaxation. The differences were significant (p <0.001*;* 0.8 ± 0.3% at 2 V to 47.1 ± 4.1% at 20 V without L-citrulline; 0.9 ± 0.3% at 2 V to 68.2 ± 4.8% at 20 V with L-citrulline). Inhibition of ASS or ASL prevented this effect of L-citrulline.

**Conclusion:**

The results indicate the presence of an L-citrulline/L-arginine cycle in the airways of rat pups. L-citrulline recycling does not play a major role under basal conditions in airways, but it has an important role under conditions of substrate limitations to NOS as a source of L-arginine, and L-citrulline supplementation reverses the impaired relaxation of airways under hyperoxic conditions.

## Background

Bronchopulmonary dysplasia (BPD) is a chronic respiratory disease that results from complications related to the lung injury during treatment of respiratory distress syndrome in premature babies. Increased airway reactivity in childhood is the most common long-term manifestation of neonatal lung injury and is pronounced when there is a history of BPD [1,2]. While a variety of pre- and postnatal inflammatory processes may contribute to its etiology, exposure of an immature respiratory tract to increased supplemental oxygen plays an important contributing role to the development of BPD [3]. Therefore, neonatal hyperoxic exposure may serve as a model for BPD in which enhanced airway reactivity is a well-known consequence. We and others have demonstrated in rat pups that exposure to high inspired oxygen is associated with many pathophysiological features of BPD, including increased contractile responses and decreased relaxant responses of airways under *in vitro* and *in vivo* conditions using trachea and lung parenchymal tissues, as well as changes in lung morphology [4-11].

Nitric oxide (NO) is a potent relaxant of airway smooth muscle (ASM) [12,13]. Exposure of rat pups to high oxygen concentration has been shown to impair relaxant responses of the tracheal smooth muscle (TSM) [8] and the distal airways using lung parenchymal tissue [4,5]. The decreased NO production was implicated in hyperoxia-induced impairment of relaxation of airways NO-cGMP-dependent mechanism (14). In order to compensate for the decrease of endogenous NO in neonatal care, exogenous NO is frequently used to prevent the adverse effects of neonatal hyperoxia [14-16]. Recent randomized human trials suggest that inhaled NO (iNO) decreases the incidence of BPD in subsets of premature babies [3,17,18]. However, inconsistent efficacy [19], complexity of iNO delivery in nonintubated patients, and high cost provide rationales for efficacious alternatives to iNO.

**Figure 1:**
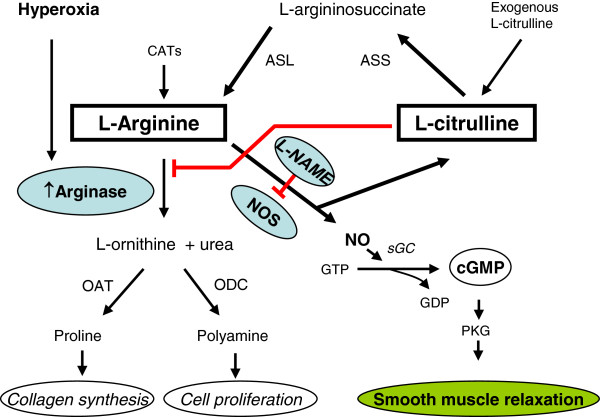
**Schematic presentation of the role of L-citrulline/L-arginine cycle in the regulation of airway function.** The diagram integrates physiologic, biochemical and molecular mechanisms whereby neonatal hyperoxic exposure impairs NO-cGMP signaling and resultant airway smooth muscle relaxation. The L-arginine is a common substrate for NOS and arginase. Hyperoxia increases the lung arginase activity that reduces the bioavailability of L-arginine to NOS. L-citrulline is recycled to L-arginine through ASS and ASL enzymes. It is proposed that L-citrulline competes with NOS blockers (like L-NAME). However, it blocks arginase activity in hyperoxic conditions and thus increases the bioavailibility of L-arginine. The red lines with bars indicate inhibition. ASS, argininosuccinate synthase; ASL, argininosuccinate lyase; CATs, cationic amino acid transporters; cGMP, cyclic guanosine monophosphate; GDP, guanosine diphosphate; GTP, guanosine triphosphate; L-NAME, N^ω^-nitro-L-arginine methyl ester; NO, nitric oxide; NOS, nitric oxide synthase; OAT, ornithine amino transferase; ODC, ornithine decarboxylase; PKG, protein kinase G; sGC, soluble guanylate cyclase.

As NO is produced from L-arginine via NO synthase (NOS) [20], bioavailability of L-arginine plays a key role in the production of NO and on lung function. In most cells, L-arginine requirements are met primarily by the uptake of extracellular L-arginine via specific cationic amino acid transporters (system y^+^) [21]. However, in NO-producing cells, L-arginine may also be obtained through the L-citrulline/L-arginine cycle [22]. Through this cycle, L-citrulline can be recycled as L-arginine to the NOS pathway by the successive action of argininosuccinate synthase (ASS), which converts L-citrulline and L-aspartate into L-argininosuccinate, and then the argininosuccinate lyase (ASL) hydrolyses L-argininosuccinate into L-arginine and fumarate [22-25], (Figure 1). Some authors suggest that L-citrulline might be an indirect precursor of NO in NO-synthesizing cells [26]. L-citrulline has been shown to restore impaired relaxation of airways after early asthmatic reaction [27]. Also in other tissues, including the opossum internal anal sphincter and rat gastric fundus, it has been shown that the L-citrulline/L-arginine cycle is involved in inhibitory nonadrenergic-noncholinergic nerve (iNANC)- mediated smooth muscle relaxation, under conditions of limited L-arginine availability to nNOS, which is induced by competitive inhibitors of the enzyme [28-31]. Furthermore, it was demonstrated that L-citrulline prevents O_2_-inducing alveolar damage and alters angiogenesis in developing lungs [11]. However, there are no studies showing the role of the L-citrulline/L-arginine cycle on hyperoxia-induced impairment of ASM relaxation. The study of L-citrulline becomes even more important in hyperoxic condition because, due to the increased arginase activity [32], conditions of limited L-arginine availability for NOS are created. Therefore, this study was undertaken to test the hypothesis that the L-citrulline/L-arginine cycle is active in rat pup airways and that L-citrulline supplementation will reverse hyperoxia-induced impaired relaxation of ASM.

## Methods

### Animals and tissue preparation

Sprague–Dawley rat pups were used for these experiments. On the 5^th^ day of life (P5), weight-matched rat pups from different litters were randomly mixed and assigned to either hyperoxic (*n* = 32) or room air (*n* = 43) groups. We have shown earlier that hyperoxic exposure for seven days starting from day 5 shows BPD-like symptoms in rat pups [4]. Therefore, the pups were exposed to hyperoxia (>95% O_2_) or room air for seven days. Hyperoxic animals were housed in a Plexiglas chamber and exposed to a continuous flow of O_2_ (2 L/min), whereas control animals were kept in a commercial rat cage in room air. Mothers were rotated each day between room air and hyperoxic groups to protect them from the toxicity of a constant hyperoxic exposure. Oxygen concentration was monitored continuously via oxygen analyzer (Pro O_2_ Nuvair, Oxnard, CA). In one set of experiments, the animals were injected intraperitoneally (i.p.) with 200 mg/kg/day L-citrulline during hyperoxic exposure. Control animals were injected with the same volume of saline. No differences in weight were observed between the compared groups.

Animals were euthanized by asphyxiation in CO_2_ on day 12 of postnatal life (P12). The trachea was removed and prepared with the intact epithelium, free of serosal connective tissue, in an ice-cold oxygenated Krebs-Henseleit (KH) solution (concentration in mM: 118.2 NaCl, 25 NaHCO_3_, 4.6 KCl, 1.2 KH_2_PO_4_, 1.2 MgSO_4_, 2.5 CaCl_2,_ and 10% dextrose, pH = 7.4). A cylindrical airway segment of 3-mm length was isolated from the mid-portion of the tracheas of each animal, and then suspended between a stainless hook at the bottom of the organ bath and a force displacement transducer (FT 03 C, Grass Instruments, Quincy, MA). TSM tension was measured by a Power Lab/4SP AD Instruments, monitored and recorded using Chart 4.0 software. An initial load of 0.3 g was used, and then tissues were allowed to equilibrate for 40 min in the organ baths containing KH solution (20 ml) at 37°C. The solution was continuously aerosolized with a gas mixture of 95% O_2_ and 5% CO_2_. All protocols described in this study were approved by the Internal Animal Use and Care Committees of the University of Prishtina and Case Western Reserve University.

### Electrical field stimulation-induced relaxation experiments

After equilibration, a cumulative concentration-response curve was made to find the concentration of bethanechol that elicited 50–75% of maximal response in TSM (100 μM bethanechol). Incremental electrical field stimulation (EFS) was applied for 10s at 2-min intervals to induce relaxation through platinum electrodes to preconstricted TSM at various voltages (2–20 V, AC). The relaxation of cylinders after EFS was expressed as a percentage of the total preconstricted state for each cylinder [4].

An NOS inhibitor – N^ω^-nitro-L-arginine methyl ester (L-NAME; 100 μM) and L-citrulline (2 mM) were applied to the organ bath 30 min prior to the addition of bethanechol, while an ASS inhibitor–α-methyl-D,L-aspartate (α-MDLA; 1 mM) and an ASL inhibitor–succinate (1 mM) were applied 40 min before bethanecol.

### Statistical analysis

The results are expressed as mean ± SEM. Statistical significance was determined by Two-way ANOVA with repeated measurements. To analyze the differences in treatments at different voltage settings between groups and within a group, a *post-hoc* Tukey–Kramer multiple comparison test was used. In all cases, p < 0.05 was considered statistically significant.

## Results

### Effect of hyperoxia on ASM relaxation

**Figure 2:**
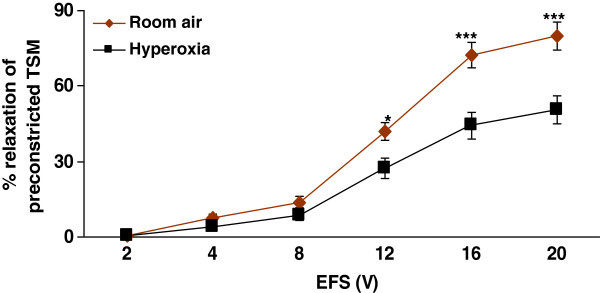
**Effect of hyperoxia on EFS-induced TSM relaxation:** EFS-induced relaxation of TSM was impaired in tissues obtained from rat pups exposed to hyperoxia as compared to those from room air exposed rat pups. *Room air vs. hyperoxic rat pups (*n* = 10 for each group). *p < 0.05; ***p < 0.001.

Consistent with our previous studies, EFS-induced relaxation increased with the increasing voltages and hyperoxia significantly reduced the EFS-induced relaxation of preconstricted TSM (*n* = 10) as compared to room air (p < 0.001; n = 10). The relaxant responses in the hyperoxic group ranged from 0.5 ± 0.1% at 2 V to 50.6 ± 5.7% at 20 V while those in the control group ranged from 0.7 ± 0.2% at 2 V to 80.0 ± 5.6% at 20 V) (Figure 2). The differences became significant at ≥ 12 V (Figure 2). Overall there was a significant difference between incremental voltages within the groups (p < 0.01).

### Role of L-citrulline, α-MDLA and succinate on EFS-induced TSM relaxation in room air-exposed animals

**Figure 3:**
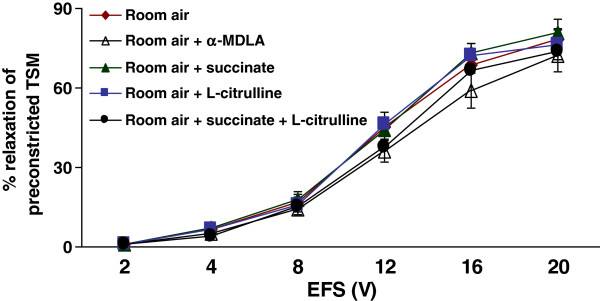
**Role of L-citrulline, α-MDLA, and succinate on EFS-induced relaxation of TSM of rat pups exposed to room air:** No significant effect of L-citrulline, α-MDLA and L-succinate individually or in combination was observed in TSM of room air exposed rat pups (*n* = 7 for each protocol of the experiments).

To study the role of the L-citrulline/L-arginine cycle under basal conditions, the tissues were incubated with L-citrulline (2 mM), or α-MDLA (1 mM), or succinate (1 mM), or in combination of L-citrulline with succinate. There was no effect of L-citrulline on the EFS-induced relaxation of preconstricted TSM of rat pups when compared to control responses (*n* = 7 per group). The α-MDLA, succinate or combination of L-citrulline and succinate did not produce any significant effect on EFS-induced relaxant responses (*n* = 7 per group). The relaxation values of TSM obtained from control tissues ranged from 1.1 ± 0.4% at 2 V to 78.1 ± 4.0% at 20 V (Figure 3). These data indicate that the L-citrulline/L-arginine cycle does not play a major role in the airway relaxation of rat pups under basal conditions (Figure 3).

### Effect of L-citrulline, α-MDLA, and succinate on TSM relaxation after NOS blockage in room air-exposed animals

**Figure 4:**
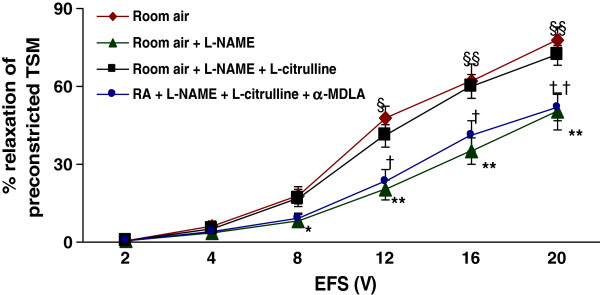
**Effect of L-citrulline and α-MDLA on TSM relaxation after NOS blockage:** EFS-induced relaxation of TSM preparations obtained from room air-exposed rat pups was significanlty (p < 0.01) reduced by the addition of 100 μM L-NAME. The decrease in relaxation due to L-NAME was returned to normal levels by the addition of L-citrulline (2 mM). The argininosuccinate synthase (ASS) inhibitor – α-MDLA (1 mM) prevented the capability of L-citrulline to recover the diminished relaxation responses due to L-NAME. * *Room air + L-NAME vs. Room air;* § *Room air + L-NAME + L-citrulline vs. Room air + L-NAME;* † *Room air + L-NAME + L-citrulline +* α-MDLA *vs. Room air + L-NAME + L-citrulline.* **p < 0.01; §p < 0.05; §§p < 0.01; †p < 0.05; ††p < 0.01. (*n* = 8 for each protocol of the experiments).

**Figure 5:**
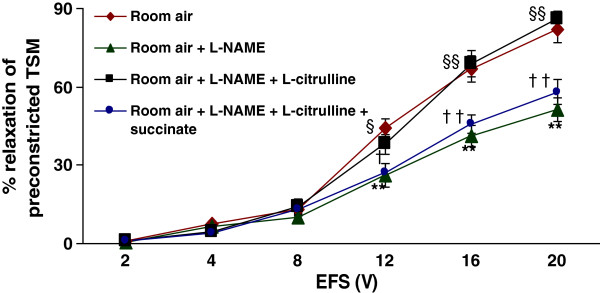
**Effect of L-citrulline and succinate on TSM relaxation after NOS blockage:** EFS-induced relaxtion of TSM preparations obtained from room air-exposed rat pups was significanlty (p < 0.01) reduced by the addition of 100 μM L-NAME. The decrease in relaxation due to L-NAME was returned to normal levels by the addition of L-citrulline (2 mM). The argininosuccinate lyase (ASL) inhibitor – succinate (1 mM) prevented the capability of L-citulline to recover the diminished relaxation responces due to L-NAME. * *Room air + L-NAME vs. Room air;* § *Room air + L-NAME + L-citrulline vs. Room air + L-NAME;* † *Room air + L-NAME + L-citrulline + succinate vs. Room air + L-NAME + L-citrulline.* **p < 0.01; §p < 0.05; §§p < 0.01; †p < 0.05; ††p < 0.01. (*n* = 8 for each protocol of the experiments).

To study the effect of L-citrulline recycling under conditions of reduced utilization of L-arginine by NOS, tracheal rings obtained from the room air-exposed animals (*n* = 8) were preincubated with an NOS inhibitor–L-NAME (100 μM), and then constricted using bethanecol. L-NAME significantly reduced (p *< 0.01)* the EFS-induced relaxation of TSM compared to the control responses, particularly at higher voltages (8–20 V; p < 0.01*),* (Figure 4). The data ranged from 0.7 ± 0.2% at 2 V to 50.5 ± 7.2% at 20 V, whereas in the control responses, the data ranged from 0.5 ± 0.2% at 2 V to 78.0 ± 4.9% at 20 V. Incubation of tissues with L-citrulline (2 mM) reversed the inhibition of EFS-induced relaxation of TSM produced by L-NAME (p < 0.01), indicating a critical role of L-citrulline under limiting conditions of NO production. These data ranged from 0.6 ± 0.1% at 2 V to 72.1 ± 4.0% at 20 V, (Figure 4). α-MDLA (1 mM) prevented the capability of L-citrulline to restore diminished relaxant responses of TSM due to L-NAME. These differences were significant (p < 0.01*)* when compared to responses obtained from tissues coincubated in L-NAME plus L-citrulline. The data ranged from 0.4 ± 0.2% at 2 V to 52.3 ± 5.2% at 20 V. Similarly, succinate significantly (p < 0.01) prevented the effect of L-citrulline to restore the reduced relaxant responses of TSM due to L-NAME. The data ranged from 0.8 ± 0.4% at 2 V to 57.9 ± 4.7% at 20 V (Figure 5).

### Effect of L-citrulline, α-MDLA and succinate on TSM relaxation in hyperoxia-exposed rat pups

**Figure 6:**
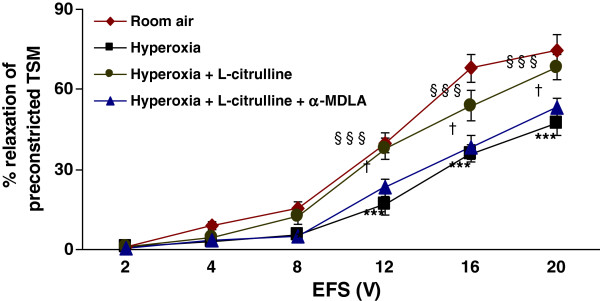
**Effect of L-citrulline and inhibition of argininosuccinate synthase (ASS) on TSM relaxation in hyperoxia-exposed rat pups:** L-citrulline supplementation (2 mM) reversed the hyperoxia-induced impairment of relaxation of TSM (p < 0.001). The effect of the L-citrulline was lost when TSM was incubated in the presence of argininosuccinate synthase (ASS) inhibitor, α-MDLA (1 mM) (p < 0.05). *Hyperoxia vs. Room air; §*Hyperoxia + L-citrulline vs. Hyperoxia*; †*Hyperoxia + L-citrulline + α-MDLA vs. Hyperoxia + L-citrulline*. *p < 0.05; ***p < 0.001; §§§p < 0.001; †p < 0.05 (*n* = 7 for each protocol of the experiments).

**Figure 7:**
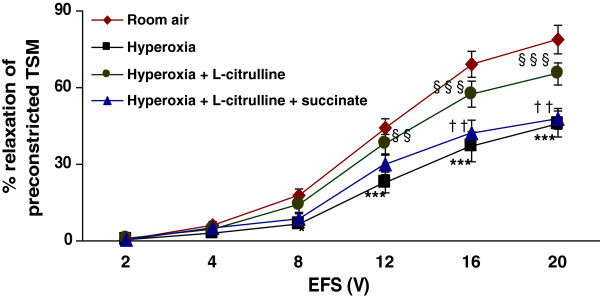
**Effect of L-citrulline and inhibition of argininosuccinate lyase (ASL) on TSM relaxation in hyperoxia-exposed rat pups:** The addition of 2 mM L-citrulline reversed the hyperoxia-induced impairment of relaxation of TSM (p < 0.001). The effect of the L-citrulline was lost when TSM was incubated in the presence of argininosuccinate lyase (ASL) inhibitor, L-succinate (1 mM) (p < 0.01*)*. *Hyperoxia vs. Room air; §*Hyperoxia + L-citrulline vs. Hyperoxia*; †*Hyperoxia + L-citrulline + succinate vs. Hyperoxia + L-citrulline*. *p < 0.05; ***p < 0.001; §§p < 0.01*;* §§§p < 0.001; †p < 0.05; ††p < 0.01*(n* = 7 for each protocol of the experiments).

As shown above, hyperoxia impaired the EFS-induced relaxation of rat pup TSM (p < 0.001) as compared to room air-exposed animals (*n* = 7 per group). The data ranged from 0.8 ± 0.3% at 2 V to 47.1 ± 4.1% at 20 V in hyperoxic animals and 0.9 ± 0.2% at 2 V to 74.8 ± 5.6% at 20 V in room air animals. L-citrulline supplementation (2 mM) reversed the hyperoxia-induced impaired relaxation (p < 0.001). The data ranged 0.9 ± 0.3% at 2 V to 68.2 ± 4.8% at 20 V (in hyperoxia + L-citrulline group) The differences were significant at higher voltages ≥ 8 V (p < 0.001). Both the ASS inhibitor, α-MDLA (1 mM) and the ASL inhibitor, succinate (1 mM) prevented L-citrulline from reversing the hyperoxia-induced impairment of relaxant responses (Figure 6 and Figure 7). The data ranged from 0.6 ± 0.1% at 2 V to 53.1 ± 3.5% at 20 V (in the presence of α-MDLA) and from 0.7 ± 0.2% at 2 V to 47.9 ± 4.1% at 20 V (in the presence of succinate), respectively.

### Intraperitoneal supplementation of L-citrulline reversed the hyperoxia-induced impaired relaxation

**Figure 8:**
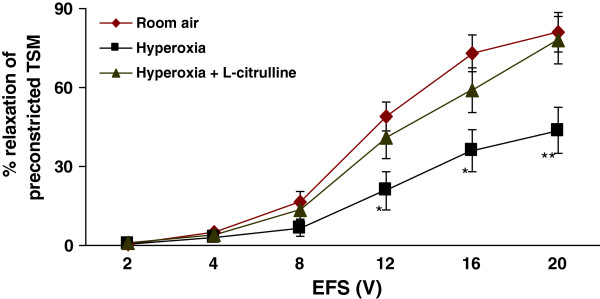
**Effect of* in vivo *L-citrulline supplementation on TSM relaxation in hyperoxia-exposed rat pups:*** In vivo* supplementation of rat pups with L-citrulline (200 mg/kg/day) reversed the hyperoxia-induced impairment of relaxant responses of TSM in the hyperoxic animals (p < 0.01). * *Hyperoxia + L-citrulline vs. Hyperoxia*; *p < 0.05; **p < 0.01 (*n* = 4 for each protocol of the experiments).

As shown in Figure 8*i.p.* supplementation of rat pups with L-citrulline (200 mg/kg/day) reversed the impaired relaxation in hyperoxic animals to normal levels compared to hyperoxic controls (p < 0.01). The differences were significant at higher voltages (≥ 12 V).

## Discussion

Prior research by us has shown that the long-term exposure of hyperoxia impairs airway relaxation [4,32] and enhances airway contraction in neonatal rat lung [5]. In this study we have shown that hyperoxia decreases EFS-induced relaxant responses of ASM. Both of these processes complement each other in increasing airway hyperreactivity. Hyperoxia increases arginase activity, which limits the availability of L-arginine to NOS [4]. Limitation of L-arginine, being a common substrate for arginase and NOS, disrupts the NO/cGMP signaling pathway, resulting in impaired relaxation [4,32]. In our recent publication we have shown that production of NO in ASM cells is reduced in hyperoxia-exposed rat pups [32]. In a lung cell, L-arginine is used as a substrate for NOS and is obtained from different sources for the production of NO, including the recycling of L-citrulline [31]. Maarsingh *et al.*[27] have shown the existence of an L-citrulline/L-arginine cycle in TSM. We have observed that the supplementation of L–citrulline reversed the hyperoxia-induced impaired relaxation in both *in vivo* (i.p. administration to animals) and *in vitro* (addition to organ bath) conditions. The *in vitro* study indicates that in rat TSM, there is an active L-citrulline/L-arginine cycle that pro-duces L-arginine. Since the systemic administration of L-citrulline via i.p. route is also effective, we assume that, unlike L-arginine, L-citrulline is not taken up by the liver from the portal circulation, but is metabolized in the kidney to L-arginine that increases the plasma concentration of L-arginine. For this reason, oral or systemic administration of L-citrulline could be a better substitute for L-arginine as it bypasses the liver [22].

The inhibition of the enzymes (ASL and ASS) involved in the recycling pathway of L-citrulline to L-arginine did not affect EFS-induced relaxation of TSM under basal conditions, indicating that L-arginine in basal conditions is not a limiting substrate. Furthermore, the supplementation of TSM with exogenous L-citrulline did not affect EFS-induced relaxation, indicating that the supply of L-arginine for NOS through L-citrulline cycling is not an important source for NOS substrate under these conditions. However, this pathway appears to play a key role once the NOS enzyme is blocked. Interestingly, L-citrulline supplementation fully restored the reduced relaxation caused by the blockage of NOS under basal conditions. The effect of L-citrulline was more prominent particularly at higher voltages when an extra supply of NO is required to compensate the changes. This observation suggests that L-citrulline competes with the NOS blocker (L-NAME) reversing its inhibitory effects. These findings are comparable with other studies performed in the tracheal rings of a guinea pig model of asthma and human ASM using single or multiple frequencies of EFS [3,27], where the inhibitory effect of NOS inhibitor, N^ω^-nitro-L-arginine, on NO-mediated iNANC relaxation was restored by L-citrulline as well as L-arginine [33]. In addition, the inhibition of ASL reversed this beneficial effect of L-citrulline supplementation, indicating the presence of an active L-citrulline/L-arginine cycle in the airways of rat pups under conditions of substrate limitation for NO production. Similar results were shown in the tracheal rings of guinea pigs [27].

In asthmatic patients, a decrease in plasma L-arginine levels and an increase in serum arginase activity was observed [34]. Similarly, in the experimental BPD in neonatal rats, the plasma level of L-arginine was found to have decreased after hyperoxic exposure of animals [11], while arginase protein expression was upregulated in the airway epithelium, further depriving the adjacent ASM for L-arginine substrate. Furthermore, Malleske *et al*. [35] showed that hyperoxia diminishes NO production in mice because of the increase in hepatic arginase activity. L-citrulline also plays a key role in the reversal of impaired relaxation during hyperoxia that creates a L-arginine limiting condition. L-citrulline has been reported to be a noncompetitive inhibitor of arginase [36]. Therefore, in hyperoxic conditions, L-citrulline appears to work through arginase inhibition rather than competition with NOS inhibitors as discussed above.

In a rat pup model of BPD, L-arginine becomes a limiting substrate and disrupts the NO-cGMP signaling pathway, due to the hyperoxia-induced upregulation of arginase activity. The disruption of NO-cGMP signaling reduces the relaxation of airways as a consequence [4,32]. An increase of arginase activity has been linked to airway hyperreactivity under different conditions in different animal models, such as rat pups, mice, and guinea pigs, as well as in humans [34,35,37-39]. In the perfused trachea of guinea pigs, it was demonstrated that increased arginase activity contributes to the airway hyperresponsiveness after early asthmatic reaction and deficiency of cNOS-derived NO [40]. Marsingh *et al*. [41] have shown that the increase of arginase activity after early asthmatic reaction in guinea pigs impairs the neuronal nitric oxide-mediated relaxation of ASM. Therefore, in this study the role of L-citrulline recycling in the airways under *in vitro* and *in vivo* conditions of TSM obtained from hyperoxia exposed animals was studied. Hyperoxia-induced impairment of the relaxant responses of TSM of rat pups was reversed by L-citrulline supplementation under both *in vitro* and *in vivo* conditions, indicating the role of L-citrulline/L-arginine recycling enzymes in hyperoxic conditions. In order to demonstrate that the recovery of the impaired relaxation of TSM was due to recycling of L-citrulline into L-arginine, the ASS and ASL were inhibited using specific inhibitors. Interestingly, the protective effect of L-citrulline in hyperoxic TSM was lost by the inhibition of ASS, as well as ASL activity, further confirming the role of the L-citrulline/L-arginine cycle and the substrate limitation to NOS. Maarsingh *et al*. [27] also have found that exogenous L-citrulline fully reversed the impaired iNANC relaxation in the guinea pig model of asthma. We have shown that after hyperoxic exposure, arginase activity increased in the experimental BPD model [4,32]. This model demonstrates that the raised levels of arginase activity, which compete with NOS for common substrate, contribute to the impairment of relaxation in most distal airways because of the decrease of NO production [4,32].

Since it has been shown that the limitation of L-arginine availability to NOS is involved in pathogenesis of several diseases involving NO deficiencies, L-arginine supplementation to the animals has been used to treat these disorders. We have observed that the supplementation of 300 mg/kg of L-arginine single dose given daily for seven days through the intraperitoneal route reversed the impaired relaxant responses in rat pups [32]. Although L-arginine supplementation in rat pups exposed to hyperoxia seems to normalize impaired relaxation of lung parenchymal strips, it further increased arginase activity, thus reducing the effectiveness of L-arginine therapy. In addition, oral L-arginine administration failed to reduce airway hyperresponsiveness in a murine model of allergic asthma [42] or in asthmatic patients [43]. This failure might be because of the presystemic elimination of L-arginine by increased intestinal or hepatic arginase activity [44].

In contrast, L-citrulline is not taken up by the liver, but is metabolized predominantly in the kidney to L-arginine and could be considered as a masked precursor of L-arginine bypassing the liver [24], thus providing plenty of substrate for NO-cGMP signaling pathway [45]. The benefits of oral administration of L-citrulline have been shown in many diseases. Oral administration of L-citrulline in patients suffering from sickle cell disease raised the decreased plasma levels of L-arginine without any toxicity [46]. Furthermore, L-citrulline decreased pulmonary hypertension after surgery in patients with congenital heart disease [47], and it also reduced blood pressure response to cold stress [48]. Recently, Cormio *et al*. [49] have shown that oral L-citrulline supplementation improves erection hardness in men with mild erectile dysfunction. Another study revealed that supplementation of L-citrulline prevents hyperoxia-induced lung injury in newborn rats [11]. L-citrulline also ameliorates development of pulmonary hypertension and increases NO production in piglets exposed to chronic hypoxia [50]. These studies provide evidence that support the use of L-citrulline as a therapy in diseases characterized by NO deficiency.

The animal model used in this study has proven quite successful to characterize the role of the L-citrulline/L-arginine cycle under normal and limiting conditions of NOS substrate. We recognize that this model under *in vitro* conditions has limitations, as it is isolated from systemic circulation and lacks the connections from central nervous system, which also affect the physiology of airways.

## Conclusion

This study demonstrates that the prolonged hyperoxic exposure to new born rats causes impaired relaxation of tracheal smooth muscle. Supplementation of L-citrulline *in vitro* and *in vivo* reverses hyperoxia-induced attenuated relaxant responses of rat pup TSM in L-arginine limiting conditions. However, L–citrulline has no effect on relaxation in basal conditions when L-arginine is not a limiting factor. The evidence originated from this study suggests that supplementation with L-citrulline may have higher potential than L-arginine as a therapeutic use for preterm infants at risk for increased airway reactivity.

## Abbreviations

ASM: Airway smooth muscle; α-MDLA: α-methyl-D,L-aspartate; ASL: Argininosuccinate lyase; ASS: Argininosuccinate synthase; BPD: Bronchopulmonary dysplasia; EFS: Electrical field stimulation; iNANC: Inhibitory non-adrenergic non-cholinergic; KH: Krebs-Henseleit; L-NAME: N^ω^-nitro-L-arginine methyl ester; nNOS: Neuronal; NOS; NO: Nitric oxide; NOS: Nitric oxide synthase; TSM: Tracheal smooth muscle.

## Competing interest

The authors do not have any competing interests.

## Authors’ contributions

RBS designed and coordinated the study, performed a major part of the experiments, and drafted the manuscript. SIAZ participated in designing the study, carried out a part of experiments, and contributed to finalizing the manuscript. MM substantially participated in experiments and revised the manuscript. HS performed the *in vivo* experiments. ZI assisted in the experiments, analyzed the data, supervised the statistics, and contributed to the manuscript. IG contributed to the study design, data interpretation, and final revision of the manuscript. AL advised during the experiments and contributed to finalizing the manuscript. MJ conceived the study and participated in its design and direction, as well as preparing the manuscript. All authors read and approved the final draft of the manuscript.

## Aknowledgements

This study was supported partially by the Ministry of Health of the Republic of Kosovo [MSH-90300576/11] and an IKARIA grant to SIAZ.
